# A data-adaptive methods in detecting exogenous methyltransferase accessible chromatin in human genome using nanopore sequencing

**DOI:** 10.1093/bioinformatics/btae206

**Published:** 2024-04-13

**Authors:** Kailing Tu, Xuemei Li, Qilin Zhang, Wei Huang, Dan Xie

**Affiliations:** National Frontier Center of Disease Molecular Network, State Key Laboratory of Biotherapy, West China Hospital, Sichuan University, Chengdu, Sichuan 610000, China; National Frontier Center of Disease Molecular Network, State Key Laboratory of Biotherapy, West China Hospital, Sichuan University, Chengdu, Sichuan 610000, China; National Frontier Center of Disease Molecular Network, State Key Laboratory of Biotherapy, West China Hospital, Sichuan University, Chengdu, Sichuan 610000, China; School of Mathematics and Statistics, Key Laboratory for Applied Statistics of the Ministry of Education, Northeast Normal University, Changchun 130024, China; National Frontier Center of Disease Molecular Network, State Key Laboratory of Biotherapy, West China Hospital, Sichuan University, Chengdu, Sichuan 610000, China

## Abstract

**Motivation:**

Identifying chromatin accessibility is one of the key steps in studying the regulation of eukaryotic genomes. The combination of exogenous methyltransferase and nanopore sequencing provides an strategy to identify open chromatin over long genomic ranges at the single-molecule scale. However, endogenous methylation, non-open-chromatin-specific exogenous methylation and base-calling errors limit the accuracy and hinders its application to complex genomes.

**Results:**

We systematically evaluated the impact of these three influence factors, and developed a model-based computational method, methyltransferase accessible genome region finder (MAGNIFIER), to address the issues. By incorporating control data, MAGNIFIER attenuates the three influence factors with data-adaptive comparison strategy. We demonstrate that MAGNIFIER is not only sensitive to identify the open chromatin with much improved accuracy, but also able to detect the chromatin accessibility of repetitive regions that are missed by NGS-based methods. By incorporating long-read RNA-seq data, we revealed the association between the accessible Alu elements and non-classic gene isoforms.

**Availability and implementation:**

Freely available on web at https://github.com/Goatofmountain/MAGNIFIER.

## 1 Introduction

Identifying open chromatin is one of the basic steps for predicting regulatory elements and understanding the functional organization of the genome (Klemm *et al.* 2019). Current established methods for open chromatin research mainly rely on next-generation sequencing (NGS) and can be divided into two types. The “Digestion-Enrichment” methods, including MNase-seq (Mieczkowski *et al.* 2016), DNaseI-seq (Crawford *et al.* 2006, Sabo *et al.* 2006), and ATAC-seq (Buenrostro *et al.* 2013), identify open chromatin by digesting DNA with nucleic acid cleavage enzymes and enriching them for sequencing. The “exogenous-labeling” methods, such as NOMe-seq, labeling open chromatin with exogenous methylation and detect them through bisulfite sequencing (Kelly *et al.* 2012). However, limited by the length of reads, these NGS-based methods suffer from low detection power of the repeat elements like transposon elements (TE) and tandem duplication (TD).

Benefiting from direct detection of base modifications, nanopore-sequencing-based “exogenous labeling” methods have been developed to detect the open chromatin in endogenous-methylation-free genome, like Saccharomyces cerevisiae. However, when applying these methods on complex genomes such as human, endogenous base modification becomes a confounding factor. Existing methods circumvent this issue by utilizing less endogenously abundant epigenetic markers, such as adenine (A) (Abdulhay *et al.* 2020, Shipony *et al.* 2020, Stergachis *et al.* 2020) or the GpC base (Wang *et al.* 2019, Lee *et al.* 2020, Battaglia *et al.* 2022). These strategies reduce data resolution (Marinov *et al.* 2022) and may generate errors in genomes harboring unconventional endogenous modifications, like endogenous 6mA enriched tumor stem cells genomes (Xie *et al.* 2018). On the other hand, current base calling models still suffer from a high error rate when identifying modified bases from the raw signals (Marinov *et al.* 2022).

To address these issues, we firstly evaluated the impact of the three influence factors, including endogenous methylation, non-open-chromatin-specific exogenous methylation, and base-calling errors. Secondly, we present a hierarchical latent variable model, which jointly modeled the raw signal of nanopore sequencing from both control DNA and methyltransferase-treated DNA from the same sample. Based on the model, we developed a data-adaptive exogenous methylation detection method that allowed us to minimize the influence of the factors and evaluate chromatin accessibility across the genome. Using ATAC-seq data as standard, this new model-based strategy achieved significantly higher sensitivity and specificity than the existing nanopore sequencing based “exogenous labeling” methods. Furthermore, applying our method on K562 genome, we identified accessible elements on the repeat sequence of human genome, which were ignored by next-generation-based open chromatin detection methods. By incorporating long-read RNA-seq data, we discovered the potential role of accessible Alu elements to generate new gene isoforms.

## 2 Materials and methods

### 2.1 Cell lines and cell culture

K562 cell line was grown in DMEM (Gibco) supplemented with 10% foetal bovine serum and 1% penicillin–streptomycin antibiotics (pen-strep). GM12878 cell line was grown in RPMI1640(Gibco) supplemented with 10% foetal bovine serum and 1% penicillin–streptomycin antibiotics(pen-strep).

### 2.2 SMAC-seq experiments

SMAC-seq experiments were performed according to a published protocol (Shipony *et al.* 2020). Briefly, 1 × 106 cells (K562/GM12878) were washed with cold 1× PBS, then resuspended in 200 μl of ice-cold nuclei lysis buffer (10 mM Tris pH 7.4, 10 mM NaCl, 3 mM MgCl2, 0.1 mM EDTA, 0.5% NP-40) and incubated on ice for 10 min. Nuclei were then centrifuged at 500 g for 5 min at 4°C, resuspended in 200 μl of cold nuclei wash buffer (10 mM Tris pH 7.4, 10 mM NaCl, 3 mM MgCl2, 0.1 mM EDTA) and centrifuged again at 500 g for 5 min at 4°C. Finally, nuclei were resuspended in 100 μl of 1× GC reaction buffer (New England Biolabs, M0227S). Nuclei were first treated with M.CviPI and EcoGII by adding 200U of M.CviPI (New England Biolabs, M0227S), 200U of EcoGII (New England Biolabs, M0603S), SAM (S-adenosylmethionine) at 0.6 mMand sucrose at 300 mM, and then incubated at 37°C for 10 min. After that, sample was treated with M.SssI by adding 60 U of M.SssI (New England Biolabs, M0226S), 128 pmol of SAM and 10 mM MgCl2, and incubated at 37°C for 10 min. The reaction was stopped by adding SDS to the final concentration of 0.2% (w/v), and high molecular weight DNA was isolated for sequencing. For control nuclei, methyltransferases were replaced with nuclease-free water in the whole reaction.

### 2.3 High molecular weight DNA isolation and long-read sequencing

HMW DNA was isolated following modified protocol of Sambrook and Russell DNA extraction (Jain *et al.* 2018). Nuclei were resuspended in 500 μl of lysis buffer [10 mM Tris-Cl pH8.0, 25 mM EDTA pH8.0, 0.5% (w/v) SDS, 20 μl of proteinase K(Qiagen)], and incubated at 56°C for 2 h with gentle mixing every 30 min. The lysate was extracted with equal volume of tris-saturated phenol using phase-lock gel light, followed with phenol: chloroform: isoamylol (25:24:1). The DNA was precipitated by adding NaCl at 200 mM, twice the volume of ice-cold ethanol, then incubated on ice for 1 h and spun in a 4°C centrifuge at 13 000 r.p.m for 20 min. The DNA pellet was washed twice with 70% ethanol and dissolved in nuclease-free water. DNA libraries were prepared using Ligation Sequencing Kit (Oxford Nanopore Technologies, SQK-LSK109) following the manufacturer’s instructions. Nanopore sequencing was carried out on R9 PromethION flowcells (Oxford Nanopore Technologies, FLO-PR0002) for up to 72 h.

### 2.4 Nanopore base calling and modification base data processing

The base modification profiles for base A, CG, and GC on human genome GRCh38 were called by megalodon (https://github.com/nanoporetech/megalodon, version 2.3.0) with model configuration file res_dna_r941_min_modbases-all-context_v001.cfg from “Rerio” model of Nanopore technologies. We also calculate the modification profile for base A with base calling model provided by Vahid Akbari et.al (Akbari *et al.* 2023). In detail, the base modification profiles for base A on human genome GRCh38 were called by megalodon(version 2.5.0) with guppy server v6.1.1 with Guppy_BaseCallingModel_r9.4.1 base calling model and Remora_6mACallingModel_r9.4.1 6 mA modification calling model from https://github.com/vahidAK/6mA_Work

The modification score for the *i*th base of read *j* in genome region *k*, $MS_{i,j,k}$, was calculated through the following conversion,

(1) $$MS_{i,j,k}=\text{log}(\frac{p_{i,j,k}}{1-p_{i,j,k}})$$

where $p_{i,j,k}$ represents for the probability of the modified base. Then the co-methylation score for local region around the *i*th mod-base was calculated based on the adjacent sites,

(2) $$Y_{i,j,k}=\frac{1}{N_{i,j,k}}\sum MS_{q,j,k}$$

where q∈[i−50,i+50]. $MS_{q,j,k}$ represents for the $N_{i,j,k}$ same type of bases from read j that are located around the center base *i*. The co-methylation score are considered as the comprehensive modification stage of the local genome in read j, which was applied as the input for further analysis.

### 2.5 Modelling per-read modification data

**Figure 1.**
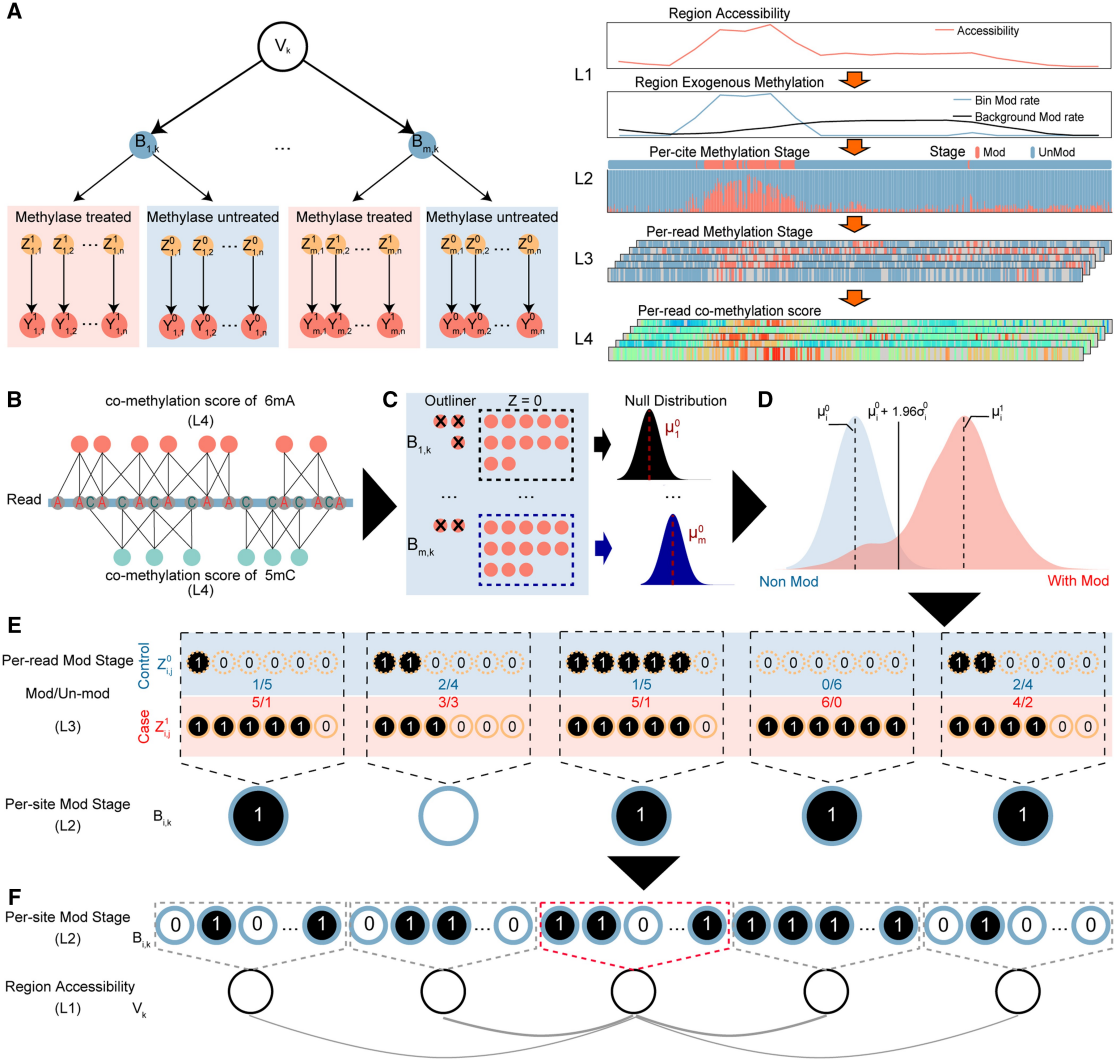
The description of the model and variable prediction. (A) Overview of the hierarchical latent variable model. (B) Schematic shows the calculation of co-methylation score. (C) Schematic shows the prediction of null distribution of each candidate base site. (D) Schematic shows the identification of loci-specific stage cutoff and the per-read status Z of each candidate base. (E) Schematic shows the prediction of per-site exogenous modification stage based on per-read status Z. Black means exogenous modified. Non-filled color means exogenous unmodified. (F) Schematic shows the calculation of chromatin accessibility.

We introduce a hierarchical latent variable model that combined both control and case data to describe the relationship between per-read co-methylation score and region accessibility as described in Fig. 1. In the observation layer(L4), for each candidate base per-read we have,

(3) $$Y_{i,j,k}^{m}|Z_{i,j,k}^{m}∼N(\Theta_{i},Z_{i,j,k}^{m}),i=1,\ldots ,S_{k}$$

where $Y_{i,j,k}^{m}$ is the co-methylation score for base *i* from read *j* of sample *m* located within region *k*. Parameter m∈{0,1} is the group label which is used to refer to the methyltransferase-treated (*m* = 1) and control group (*m* = 0). The co-methylation score follows normal distribution with parameter $\Theta_{{i,Z}_{i,j,k}^{m}}=[\mu_{i,Z_{i,j,k}^{m}},\sigma_{i,Z_{i,j,k}^{m}}]$. $Z_{i,j,k}^{m}\in \{0,1\}$ represents the exogenous modification status of locus *i* in read *j* of sample *m* belonging to region k. $Z_{i,j,k}^{m}=1$ means base *i* is methylated in read *j*. $Z_{i,j,k}^{m}=0$ represents unmethylated base status. Given $Z_{i,j,k}^{m}=0$, the co-methylation score $Y_{i,j,k}^{m}$ follows the null distribution of the base *i*, which would be inferred independently for each base *i*. Then per-read base status $Z_{i,j,k}^{m}$ (L3) is affected by the overall status of genome loci *i* within region k,$B_{i,k}^{\;}$ (L4),

(4) $$P(Z_{i,j,k}^{m}=1|B_{i,k})∼\mathrm{Bernoulli}(P_{i}^{m})$$

where $B_{i,k}^{\;}=0$ represents no exogenous modification at candidate site *i*; $B_{i,k}^{\;}=1$ represents significant exogenous modified status. The continuous exogenous modifications would enrich in open chromatin regions. The distribution of base status $B_{i,k}^{\;}$ follows another Bernoulli distribution with successive probability $P_{k}^{\;}$:

(5) $$P(B_{i,k}|V_{k})∼\mathrm{Bernoulli}(P_{k})$$

where $V_{k}\in \{0,1\}$ is the chromatin accessibility status of small genome region *k* which is set to 50 bp in length. $V_{k}=1$ represents accessible status of small region *k*, $V_{k}=0$ represents inaccessible status of region *k*.

The chromatin accessibility inference. To avoid methods with relatively high time complexity, such as those of which are applied in the Bayesian Network Inference (Murphy 2012), we take a layer-by-layer prediction strategy. In details, we first infer per-read exogenous modification stage $Z_{i,j,k}^{m}$. Reads from control sample would be considered to have similar endogenous modification status, so that for each base i, the parameters of the null distribution ($Z_{i,j,k}^{m}=0$) are directly predicted from the mean and standard deviation of observed co-methylation score of control data due to the low frequency of heterogeneous methylated data in most of base (Efron 2012) (Fig. 1B–D). In methyltransferases treated sample, reads of bases i should be a mixture of stage $Z_{i,j,k}^{m}=0$ and stage $Z_{i,j,k}^{m}=1$. Instead of inferring the modification stage $Z_{i,j,k}^{m}$ by $Y_{i,j,k}^{m}$ through EM-based mixture model. The application of null distribution inferred by control data greatly simplified the calculations. As base in methylated status would get higher score than unmethylated, bases with co-methylation score above the upper outliner of null distribution, $\mu_{i,(Z_{i,j,k}^{m}=0)}\;+\;1.96\times \sigma_{i,(Z_{i,j,k}^{m}=0)}$, would be considered as methylated base, $Z_{i,j,k}^{m}=1$. Notably when the base is endogenously methylated, the null distribution parameter $\mu_{i,(Z_{i,j,k}^{m}=0)}$ would be elevated, which facilitates our model to eliminate the influence of endogenous modification. For each candidate base site i on reference genome, reads from methyltransferases treated and control group could be regarded as two Bernoulli trails.

**Table:** 

	Num of $Z_{i,j,k}^{m}=0$	Num of $Z_{i,j,k}^{m}=1$
Treated group	$N_{i}^{m=1}-N_{i,(Z_{i,j,k}^{m}=1)}^{m=1}$	$N_{i,(Z_{i,j,k}^{m}=1)}^{m=1}$
Control group	$N_{i}^{m=0}-N_{i,(Z_{i,j,k}^{m}=1)}^{m=0}$	$N_{i,(Z_{i,j,k}^{m}=1)}^{m=0}$

Determining the overall exogenous methylated status of base i, $B_{i,k}^{\;}$, is equivalent to comparing whether the enzyme treated group have significant higher frequency of reads with exogenous modification status, $Z_{i,j,k}^{m}=1$. We applied one-tailed Fisher’s exact test to evaluate the model hypothesis and assigned candidate base sites with significantly increased exogenous modifications(*P*-value ≤ 0.01) to $B_{i,k}^{\;}=1$ (Fig. 1E). Based on the status of $B_{i,k}^{\;}$, the accessibility score of each region k (50-bp in size, and uniformly distributed across the genome) could be considered as successive probability of $V_{k}^{\;}=1$,

(7) $$\begin{array}{c} P(V_{k}=1)=\frac{\sum W(L-k)\;\times \;\mathrm{Num}(B_{i,L}=1)}{\sum W(L-k)\;\times \;\mathrm{Num}(B_{i,L}=1)\;+\;\sum W(L-k)\;\times \;\mathrm{Num}(B_{i,L}=0)}\\ L\in [k-500,k\;+\;500] \end{array}$$

The prediction is slightly different from Num(B=i,k1)Num(Bi,k=0). Considering the instability of the distribution number of candidate bases, we included the adjacent region *L*, located within 500 bp up and down stream of region *k* for weighted average based on weighted function *W*:

(8) $$W(D)=\frac{2e^{-\mu |D|}}{1+e^{-\mu |D|}}$$

where *D* represents for the distance of adjacent regions from region *k*, the decay parameter is set to $-\frac{1}{60}\text{log}(3)$ (Fig. 1F).

### 2.6 Open chromatin data acquisition and data processing

Raw ATAC-seq data of GM12878 and K562 cell lines were downloaded from ENCODE database (Consortium 2012) with accession number ENCFF415FEC, ENCFF646NWY for GM12878 and accession number ENCFF512VEZ, ENCFF987XOV for K562. scNanoATAC-seq data of both cell lines were downloaded from GEO data base with accession number GSE194022. Raw scNanoATAC-seq data of each cell were aligned to human genome GRCh38 with minimap2 (version 2.22-r1101) (Li 2018, 2021). DNase-seq defined open chromatin regions were downloaded from ENCODE database with accession number ENCFF274YGF.

### 2.7 Read end extraction of ATAC-seq and scNanoATAC-seq

Read ends of ATAC-seq data were extract through pysam package. Paired-end reads with mapping quality over 30 were extracted to form a fragment span region. The 5ʹ and 3ʹ end of each fragment were considered as the read ends. Read ends of scNanoATAC-seq data were extracted with similar method, excluding the step that involves fragment formation. Read ends of ATAC-seq and scNanoATAC-seq were overlapped with 50 bp window divided by MAGNIFIER with bedtools intersection function. For each window, the frequency of read end were calculated by the count of overlap event. The threshold for distinguishing between high and low frequency read ends was set at the top 10% quantile of the overall frequency data.

### 2.8 Data processing of Nanopore RNA-seq data

Nanopore long-read RNA sequencing data for K562 cell line was downloaded from GEO database at https://www.ncbi.nlm.nih.gov/geo/query/acc.cgi?acc=GSE205935. All long reads were aligned to human genome GRCh38 with minimap2 (version 2.22-r1101) (Li 2018, 2021) with recommended parameter (-ax splice—MD -L). Transcripts were assembled with stringtie (v2.1.7) (Kovaka *et al.* 2019) with the reference gene model from genecodev37, and parameters -L -p 50 -c 5 -s 5. New transcripts were identified with gffcompare (v0.12.6) (Pertea and Pertea 2020).

## 3 Results

### 3.1 Addressing inefficiencies in base calling models for detecting open chromatin

**Figure 2.**
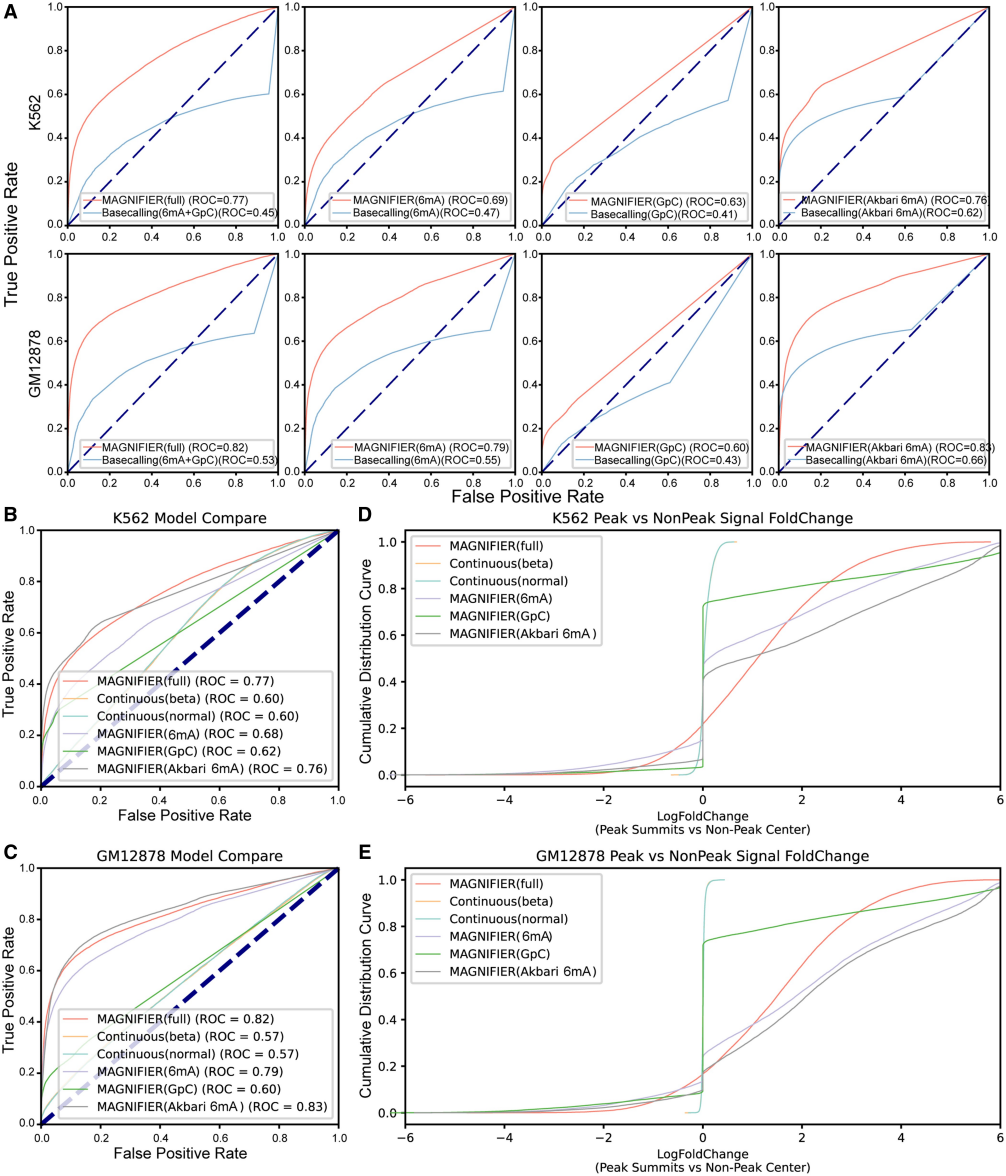
The improvement of MAGNIFIER in Open chromatin region detection. (A) ROC plots show the improvement of MAGNIFEIR compared with base calling method. In each plot, red curve means MAGNIFEIR’s ROC, blue curve means ROC curve of base calling method. (B and C) ROC plots show the ROC plot shows the improvement of MAGNIFIER compared with continuous data-adaptive model based on normal and beta distribution in K562 (B) and GM12878 (C) cell line. (D and E) Cumulative distribution curve shows the log2 fold change of model output signal between open chromatin region and nearby nonopen chromatin region of K562 (D), and GM12878 (E).

By utilizing ATAC-seq data as the benchmark, we assessed the efficacy of the MAGNIFIER model alongside other methods in determining chromatin accessibility throughout chromosome 1, focusing on identified open and nonopen chromatin regions. Compared to base calling methods, the MAGNIFIER model consistently demonstrated superior ROC values (Fig. 2A), indicating its enhanced recognition capability over conventional base calling. Base calling model inefficiency is a significant confounding factor in detecting open chromatin regions. In the output of base calling model, we found that approximately 45%–72% of data points fell into an uncertain modification status between methylated and unmethylated states (Supplementary Fig. S1A), rendering a scarcity of effective data to identify features of open chromatin (Supplementary Fig. S1B). Simple adjustments in base calling cutoffs or methylation level differences did not improve the identification of open chromatin (Supplementary Fig. S1C and D). To address this confounding factor, MAGNIFIER utilized the co-methylation scores (See Methods) for modeling and introduced a data-adaptive comparison method to enhance the resolution of open chromatin identification (Supplementary Note, Supplementary Figs S2 and S3). Even when employing the high-performance 6mA detection model proposed by Akbari *et al.* (2023) (Supplementary Fig. S1A, 3%–4% bases in uncertain modification status), MAGNIFIER still outperformed in detecting open chromatin (Fig. 2A).

### 3.2 The discrete data-adaptive strategy effectively mitigates the impact of nonopen-chromatin specific exogenous methylation confounding

For the data-adaptive comparison approach, we also established a likelihood ratio model entirely based on methylation scores (Supplementary Methods), which showed significantly lower ROC values than MAGNIFIER (Fig. 2B and C). Taking the genomic region chr1:110977600–111077600 in the K562 cell line as an example, we observed that the continuous data-adaptive model exhibited a higher incidence of false positives(Supplementary Fig. S4A). Specifically, at locus chr1:111032029, 2 out of 39 reads in the case sample showed a state of full-length high methylation (Supplementary Fig. S4B and C). These nonopen-chromatin-specific exogenous modifications, possibly originating from the DNA of dead cells, affected the fitting of the continuous data-adaptive model, leading to an overestimation of chromatin accessibility at this site (Supplementary Fig. S4D and E). In contrast, the MAGNIFIER's data-adaptive approach effectively mitigated the issue of false positives due to outlier data, ensuring robust estimation results (Supplementary Fig. S4F). However, the use of the one-tailed Fisher’s exact test method demands a substantial data volume. An evaluation of MAGNIFIER's detection performance under various down-sampling scenarios revealed a significant reduction in efficacy when the average sequencing depth dropped below 20× (Supplementary Fig. S4G).

### 3.3 MAGNIFIER overcomes endogenous methylation confounding effects

Moreover, the data-adaptive algorithm equips MAGNIFIER with the capability to use nucleotide sites featuring low endogenous methylation (with methylation levels ≤15%) as indicators for detecting open chromatin regions (Supplementary Note, Supplementary Fig. S4H). This advantage allows for the integration of markers from A, CpG, and GpC bases, leading to enhanced detection of open chromatin regions (Fig. 2D). Regions of open chromatin that may not be detectable using only 6 mA information can now be identified through the combination of various base marker data (Supplementary Note, Supplementary Fig. S5).

### 3.4 Extended detection of open chromatin regions by MAGNIFIER

**Figure 3.**
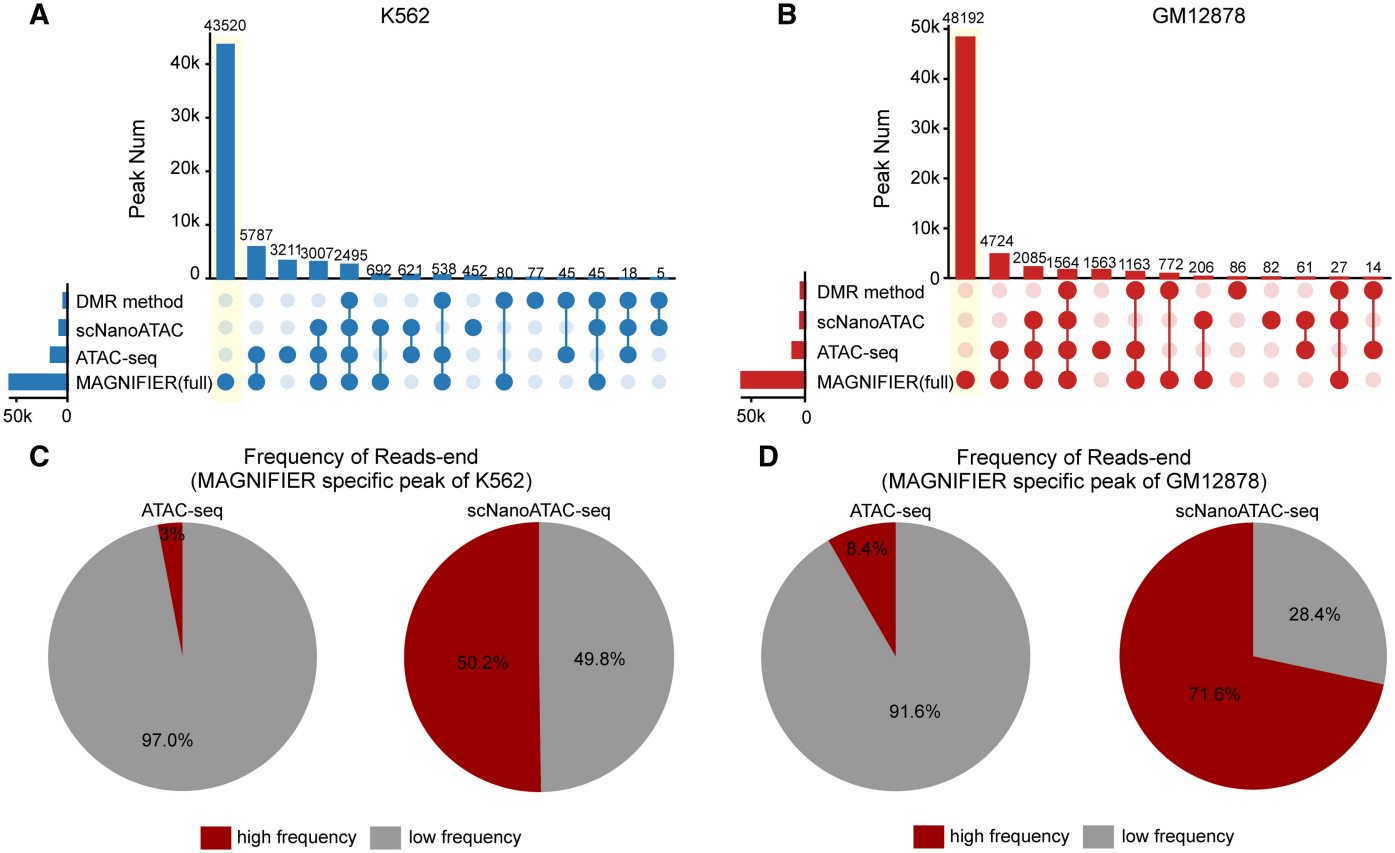
The performance of MAGNIFIER and other methods in detecting the open accessible regions. (A and B) Upset plot shows the number of open chromatin region detected by DMR method, scNanoATAC-seq, ATAC-seq, and MAGNIFIER from K562 (A) and GM12878 (B). All open chromatin region is located in chrom1. (C and D) Pie charts show the percentage of MAGNIFIER specific open chromatin regions detected from K562 (C) and GM12878 (D) with high or low ATAC (left) or scNanoATAC-seq (right) reads end frequency.

Beyond ATAC-seq, we also utilized scNanoATAC-seq (Hu *et al.* 2023) data and the open chromatin detection technique based on differential 6 mA regions (DMR method) by Akbari *et al.* (2023). for an overarching evaluation. For chromosome 1, the MAGNIFIER method uncovered most of the open chromatin regions detected by the three other methods, capturing 75.2%–85.1% of ATAC-seq peaks, 85.1%–96.4% of scNanoATAC peaks, and 95.6%–97.2% of DMR peaks (Fig. 3A and B). In addition, MAGNIFIER identified a multitude of open chromatin regions in K562 and GM12878 cell lines (Fig. 3A and B), with 50.2%–71.6% of MAGNIFIER-specific regions showing high-frequency scNanoATAC-seq read ends, but only a minimal amount (3.0%–8.4%) exhibiting high-frequency ATAC-seq read end frequencies (Methods, Fig. 3C and D), potentially related to the repetitive sequences.

**Figure 4.**
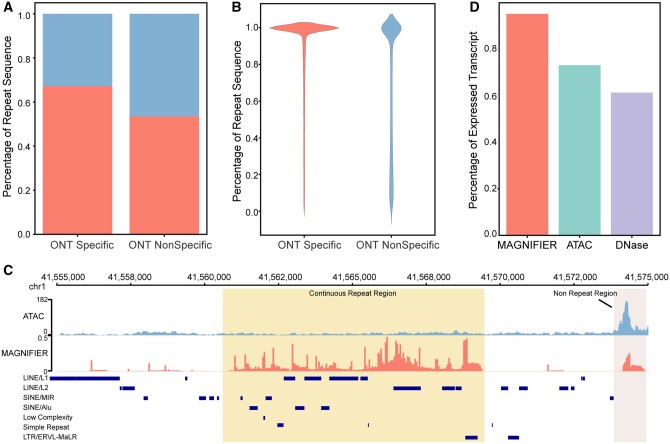
Sequence characteristics of MAGNIFIER-specific open chromatin regions. (A) Barplot shows the percentage of repeat sequence associated accessible region that specifically defined by MAGNIFIER or not. (B) Violinplot shows the distribution of repeat sequence compartment in MAGNIFIER-specific or MAGNIFIER-nonspecific open chromatin regions. (C) Snapshot of genome browser shows the chromatin accessible signal of ATAC-seq (light blue) and MAGNIFIER (red) within continuous repeat regions (labeled in light yellow) and nonrepeat regions (labeled in light grey). (D) Barplot shows the percentage of transcripts with accessible region detected around the initial site of transcripts.

In the K562 cell line, compared with the ATAC-seq-detectable open chromatin regions, the MAGNIFIER-specific open chromatin regions are more likely to intersect with repetitive sequence areas (Fig. 4A), and were more likely to consist entirely of repeat sequences (Fig. 4B and C). In contrast to nonrepeat regions, accessible repeat regions associated with ATAC-seq reads are more likely to be derived from long DNA fragments (Supplementary Fig. S6A and B). Short fragment-derived ATAC-seq reads, the primary data source for identifying open chromatin, were unable to be aligned in continuous repeat regions due to multi-alignments. However, long-read-sequencing-based technology does not suffer from this limitation. Therefore, MAGNIFIER enabled us to annotate the open chromatin located at repeat sequences around transcript start sites (TSSs) of expressed genes (Fig. 4D, Supplementary Fig. S6C and D).

### 3.5 Accessible Alu elements are associated with the regulation of gene transcripts

As mentioned above, MAGNIFIER identified additional open chromatin that were missed by other methods. Interestingly, these regions were enriched in SINE/Alu elements (Supplementary Fig. S7A), a widely distributed retrotransposon family accounting for 9%–11% of the human genome (Hancks and Kazazian 2016). In consist of the previous research (Zhang *et al.* 2019), our data showed that accessible Alu elements were enriched in introns and promoter regions (Supplementary Fig. S7B). We generated long-read RNA-seq data from K562 cell line to study the potential role of accessible Alu elements in regulating gene expression. 83.6% of exonic Alu elements were accessible in the genome (Supplementary Fig. S7C). They are mainly involved in the formation of single-exon transcripts (39.1%) as well as the first (14.3%) or last (33.5%) exon of multi-exon transcripts (Supplementary Fig. S7D and E). However, no corresponding transcripts were found for over 95% of accessible Alu elements (Supplementary Fig. S7F and G).

**Figure 5.**
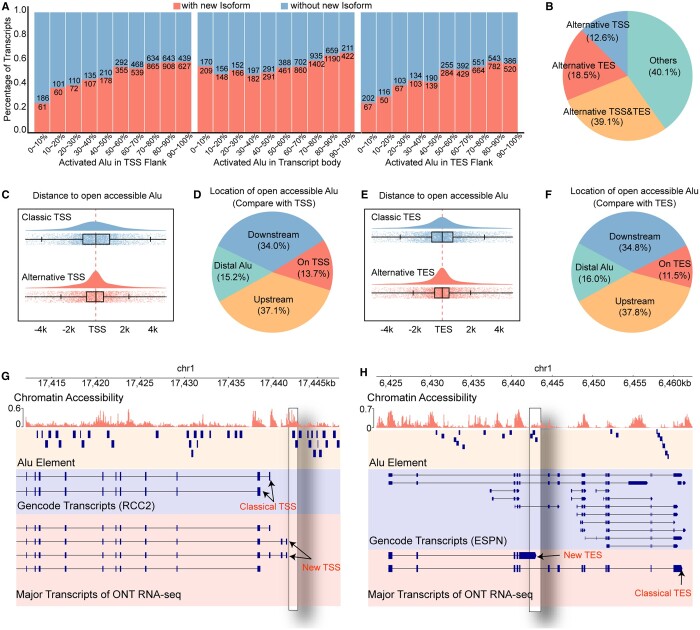
Open accessible Alu elements and new transcript. (A) Bar plot shows the percentage of genes with non-classical isoform (red) or not (blue). TSS flank is defined as genome region located within 5 kb window upstream and downstream of TSS. TES flank is defined as genome region located within 5 kb window upstream and downstream of TES. (B) Piechart shows the type of new gene isoforms. (C) Rainyplot shows the distribution of distance to the nearby open accessible Alu from classic TSS (blue) and alternative TSS (red). (D) Piechart summaries the location of open accessible Alu element to shifted TSS. (E) Rainyplot shows the distribution of distance to the nearby accessible Alu from classic TES (blue) and alternative TES (red). (F) Piechart summaries the location of accessible Alu element to shifted TES. (G) Snapshot shows the positional relationship between accessible Alu element and the TSS of new transcript of RCC2 gene. (H) Snapshot shows the positional relationship between accessible Alu element and the TES of new transcript of ESPN gene.

Notably, the proportion of new gene isoforms was positively correlated with the enrichment of accessible Alu elements around the initial (*P*-value = 9.307e−08, Spearman correlation) and terminal sites (*P*-value = 7.321e−06, Spearman correlation) of the transcripts (Fig. 5A). Approximately 59.9% of new transcripts utilized alternative transcription start sites (TSSs) or transcription end sites (TESs) (Fig. 5B). Compared with the closest classical TSS or TES annotated by GENCODE, the alternative TSSs and TESs were closer to the accessible Alu elements and mostly within 5 kb (Fig. 5C–F). The utilization of alternative TSSs or TESs imply the elongation of transcripts, such as RCC2, an oncogene associated with the proliferation (Wu *et al.* 2022), invasion (Chen *et al.* 2020), and epithelial-mesenchymal transition (Pang *et al.* 2017) of multiple cancers, or the truncation of transcripts, such as ESPN, a gene associated with cancer cell growth (Li *et al.* 2018). These phenomena indicated that the accessible Alu element affects the initiation and termination of nearby genes while the sequence itself does not necessarily transcribed.

## 4 Discussion

With the advantages of direct modified base detection, long-read exogenous modification-based chromatin accessibility detection methods, such as SMAC-seq (Shipony *et al.* 2020), and NanoMe-seq (Lee *et al.* 2020), provide new insight into open chromatin. Endogenous modification, non-open-chromatin-specific exogenous methylation, and erroneous base calling are the main bottlenecks for the widespread application of this technology in human genome. In contrast to previous research relying on binary base calling results, we proposed a data-adaptive method that infers a null distribution for each candidate locus with methylation scores from the control group and identifies the exogenous modification status in the methyltransferase-treated group. Furthermore, instead of evaluating the modification status on each individual base, our model focuses on the co-methylation status of each local region. The resolution and accuracy of open chromatin are thus significantly improved.

Benefiting from the utilization of long-read based chromatin accessibility methods, we were able to profile open chromatin in repeat genome sequences. We found that in K562 cells, the major accessible repeat sequences is enriched in Alu element, a wildly distributed in human genome (Hancks and Kazazian 2016). In contrast to the previous research focusing mainly on the expression of Alu sequences in transcripts (Zhang *et al.* 2019), our data show that most of the accessible Alu elements are not transcribed. They are potential regulators influencing the formation of new transcripts.

The current version of MAGNIFIER is not yet ready for heterogeneous tissues, as it is difficult to infer an appropriate null distribution for loci with heterogeneous modification states. A potential improvement plan would be to systematically screen these sites using a mixture model, and estimate the chromatin accessibility of each single molecule with adjacent low heterogeneous sites. Another restriction is that the current method is still unable to explore the chromatin accessibility for regions completely formed by endogenously modified bases. Adding more modification markers like N4-methylcytosine (4mC) (Timinskas *et al.* 1995) and 5-hydroxymethyluracil (Kawasaki *et al.* 2017) will help resolve this issue.

Together, the present study applied multiple exogenous modification markers and nanopore sequencing to detect chromatin accessibility in human genome for the first time. In this article, we comprehensively investigate the issues of false positives and false negatives in nanopore sequencing based chromatin accessibility detection, and demonstrated that our model outperforms other methods on both problems. Our model also provides a more flexible approach for detecting exogenous modification markers, which can theoretically be further extended to the detection of other “exogenous-labeled” markers.

## Supplementary Material

btae206_Supplementary_Data

## Data Availability

MAGNIFIER is an open source collaborative initiative available in the GitHub repository (https://github.com/Goatofmountain/MAGNIFIER). Processed data, including nanopore raw current signal data from methyltransferase-treated and control of both K562 and GM12878 cell line in this study have been deposited in the Genome Sequence Archive in BIG Data Center under accession number HRA003269 (BioProject: PRJCA012543) (Tu 2022). Raw ATAC-seq data of GM12878 and K562 cell lines were downloaded from ENCODE database with accession number ENCFF415FEC, ENCFF646NWY for GM12878 and accession number ENCFF512VEZ, ENCFF987XOV for K562. scNanoATAC-seq data of both cell lines were downloaded from GEO data base with accession number GSE194022. DNase-seq defined open chromatin regions of K562 cell line were downloaded from ENCODE database with accession number ENCFF274YGF.
