# Supplementary material for: A data-adaptive methods in detecting exogenous methyltransferase accessible chromatin in human genome using nanopore sequencing
Source: Bioinformatics. 2024 Apr 13;40(5):btae206. doi: 10.1093/bioinformatics/btae206 (PMC11256936; doi:10.1093/bioinformatics/btae206)
Supplement: btae206_Supplementary_Data [file btae206_supplementary_data.pdf]

Supplementary Methods

Full methylation outliner reads detection

To assess the prevalence of fully methylated reads, we randomly selected 100 windows with 100kb in size, across chromosome 1. We analyzed the methylation status of 6mA modifications in case data reads within these windows. Following the results from the 6mA detection model trained by Akarbri et al., reads exceeding 2kb in length with over 80% of adenine (A) site methylation scores above 1 were classified as fully methylated outlier reads.

Alternative Data-adaptive model based on continuous methylation level

In contrast to the discretized data-adaptive model used by MAGNIFIER, we developed a continuous data-adaptive model that correlates the methylation level of bases $Y_{i,j}^{m}$ with the accessibility status of chromatin sites V_i,

$$Y_{i,j}^{m} \mid V_i \sim N(\mu_i^{m}, \sigma_i^{m^2}) \tag{9}$$

Where $Y_{i,j}^{m}$ is the co-methylation score base i from read j of sample $m \in \{0,1\}$ is the group label which is used to refer to the methyltransferase-treated (m=1) and control group(m=0). The parameter of normal distribution $\mu_i^{m}, \sigma_i^{m^2}$ is estimated based on the observed data with the function stats.norm.fit function from scipy package.

When V_i located within open chromatin region, data from case and control have different distribution parameter,

$$\begin{aligned} P(V_i = 1 \mid \{Y_{i,j}^{m}\}) &\propto P(\{Y_{i,j}^{m}\} \mid V_i = 1) \times P(V_i = 1) = P(\{Y_{i,j}^{1}\} \mid V_i = 1) \times P(\{Y_{i,j}^{0}\} \mid V_i = 1) \times P(V_i = 1) \\ &= \mathrm{f}(\{Y_{i,j}^{m}\} \mid \mu_i^{1}, \sigma_i^{1^2}) \times f(\{Y_{i,j}^{m}\} \mid \mu_i^{0}, \sigma_i^{0^2}) \end{aligned} \tag{10}$$

Where $f(\bullet)$ is the likelihood function for normal distribution. When V_i located within non-open chromatin region, data from case and control have similar distribution parameter, so we use μ_i^{0}, σ_i^{0} in the likelihood calculation step,

$$\begin{aligned} P(V_i = 0 \mid \{Y_{i,j}^{m}\}) &\propto P(\{Y_{i,j}^{m}\} \mid V_i = 0) \times P(V_i = 0) = P(\{Y_{i,j}^{1}\} \mid V_i = 0) \times P(\{Y_{i,j}^{0}\} \mid V_i = 0) \times P(V_i = 0) \\ &= f(\{Y_{i,j}^{1}\} \mid \mu_i^{0}, \sigma_i^{0^2}) \times f(\{Y_{i,j}^{0}\} \mid \mu_i^{0}, \sigma_i^{0^2}) \end{aligned} \tag{11}$$

Finally we scaled the probability of $V_i = 1$,

$$P(V_i = 1) = \frac{f(\{Y_{i,j}^{1}\} \mid \mu_i^{1}, \sigma_i^{1^2}) \times f(\{Y_{i,j}^{0}\} \mid \mu_i^{0}, \sigma_i^{0^2})}{f(\{Y_{i,j}^{1}\} \mid \mu_i^{0}, \sigma_i^{0^2}) \times f(\{Y_{i,j}^{0}\} \mid \mu_i^{0}, \sigma_i^{0^2}) + f(\{Y_{i,j}^{1}\} \mid \mu_i^{1}, \sigma_i^{1^2}) \times f(\{Y_{i,j}^{0}\} \mid \mu_i^{0}, \sigma_i^{0^2})} \tag{12}$$

We also build up continuous data-adaptive model based on the probability of methylation of each base $(p_{i,j}^{m})$ provided by base calling model,

$$\mathrm{p}_{i,j}^{m} \mid V_i \sim beta(\alpha_i^{m}, \beta_i^{m}) \tag{13}$$

The parameter $\alpha_i^{m}, \beta_i^{m}$ is calculated based on the method of moments estimation,

$$\bar{x} = mean(p_{i,j}^{m})$$
$$s = std(p_{i,j}^{m})$$
$$\hat{\alpha} = \bar{x} \times (\frac{\bar{x}(1-\bar{x})}{s^2} - 1) \quad (13)$$
$$\hat{\beta} = (1 - \bar{x})(\frac{\bar{x}(1-\bar{x})}{s^2} - 1)$$

Similar to the continuous model based on normal distribution, we next calculate the probability of open and non-open chromatin status of V_i, for open chromatin,

$$P(V_i = 1 \mid \{p_{i,j}^{m}\}) \propto P(\{p_{i,j}^{m}\} \mid V_i = 1) \times P(V_i = 1) = P(\{p_{i,j}^{1}\} \mid V_i = 1) \times P(\{p_{i,j}^{0}\} \mid V_i = 1) \times P(V_i = 1)$$
$$= f(\{p_{i,j}^{1}\} \mid \alpha_i^{1}, \beta_i^{1}) \times f(\{p_{i,j}^{0}\} \mid \alpha_i^{0}, \beta_i^{0}) \quad (14)$$

For non-open chromatin,

$$P(V_i = 0 \mid \{p_{i,j}^{m}\}) \propto P(\{p_{i,j}^{m}\} \mid V_i = 0) \times P(V_i = 0) = P(\{p_{i,j}^{1}\} \mid V_i = 0) \times P(\{p_{i,j}^{0}\} \mid V_i = 0) \times P(V_i = 0)$$
$$= f(\{p_{i,j}^{1}\} \mid \alpha_i^{0}, \beta_i^{0}) \times f(\{p_{i,j}^{0}\} \mid \alpha_i^{0}, \beta_i^{0}) \quad (15)$$

and scale the probability,

$$P(V_i = 1) = \frac{f(\{p_{i,j}^1\}|\alpha_i^1,\beta_i^1)\times f(\{p_{i,j}^0\}|\alpha_i^0,\beta_i^0)}{f(\{p_{i,j}^1\}|\alpha_i^0,\beta_i^0)\times f(\{p_{i,j}^0\}|\alpha_i^0,\beta_i^0)+f(\{p_{i,j}^1\}|\alpha_i^1,\beta_i^1)\times f(\{p_{i,j}^0\}|\alpha_i^0,\beta_i^0)} \quad (12)$$

Data simulation

Methylation score data were simulated using the random.normal function from the numpy package. For the simulation of non-open chromatin-specific outlier data, control data were generated to follow a normal distribution with a mean of -0.22 and a standard deviation of 0.44, at a sequencing depth of 30X. In case data, 10% of the data points were designated as outliers with variable means within the range of [-1.5, 2.5] uniformly sampled, and a fixed standard deviation of 0.44. The distribution parameters for the remaining 27 case data points were identical to those of the control. Forty sets of outlier parameters were generated, each simulated 100 times to calculate values using the continuous data-adaptive model based on normal distribution, and one-tail Fisher's Exact test p-values were calculated using the discrete data-adaptive method employed by MAGNIFIER. For the simulation of endogenous methylation data, control data points in a state of endogenous methylation followed a normal distribution with a mean of 1.5 and a standard deviation of 0.44, while the rest followed a distribution with a mean of -0.22 and a standard deviation of 0.44.

Supplementary Note

Inefficiencies in Basecalling Models Affecting Open Chromatin Detection: In the chromosome 1 segment (chr1:167607000-167707000) of the K562 cell line, our analysis of basecalling results for all marker bases indicated a substantial inefficiency in the default basecalling model, with 45%-72% of bases left in an undetermined methylation state (Supplementary Fig.1A). This performance issue significantly affected the proportion of usable data, leading to poor resolution in chromatin accessibility regions identified by standard cutoff methods (Supplementary Fig.1B). Sole reliance on the differential analysis between case and control in default basecalling outcomes faced a high risk of false positives (Supplementary Fig.1C). Adjustments in thresholds for base modification status detection failed to effectively discern open chromatin regions through basecalling (Supplementary Fig.1D).

Utilizing co-Methylation Scores and Data-Adaptive Strategies Enhances Chromatin Accessibility Resolution: To address these issues, we constructed a data-adaptive comparison method based on the raw methylation scores provided by basecalling model. This approach entails estimating a null distribution from control data, thereby determining a dynamic threshold(Supplementary Fig.2A), which in turn facilitates the calculation of the percentage of sites in case data exceeding this threshold (UM-sites rate). The approach significantly improved signal differentiation between open and non-open chromatin regions(Supplementary Fig.2B), with 86% of recognized open chromatin regions exhibiting a higher UM-site Rate compared to adjacent non-open chromatin (Supplementary Fig.2C). Moreover, we averaged the methylation score of adjacent bases targeted by the same methyltransferase to form co-methylation score(Methods), reflecting the integrated methylation status of a local region. In this way, the continuous methylation signal associated with open chromatin will be amplified(Supplementary Fig.2D), culminating in 92.1% of open chromatin regions showcasing a superior UM-site rate relative to neighboring non-open regions (Supplementary Fig.2E). Further more, utilizing the co-methylation score extended the amount of sample size of each base (Supplementary Fig.3A). The local averaging step could impute lost data caused by incorrect base calling and strand-specific modification bases (Supplementary Fig.3B, Methods). Furthermore, the outlier data in each base were well controlled, resulting in a significantly decreased standard deviation of the co-methylation score in most bases(Supplementary Fig.3C). In enzyme untreated sample, the co-methylation scores of over 68.32% bases were normally distributed (Supplementary Fig.3D, shapiro-test $p > 0.05$). While the treated sample did not show similar characteristics (Supplementary Fig.3D, shapiro-test $p > 0.05$), over 87% of the bases located within open accessible regions showed a larger standard deviation of the co-methylation score in the treated sample than in the untreated sample (Supplementary Fig.3E), reflecting the mixture of methylated and unmethylated stages under the influence of methyltransferase. For most of bases, the co-methylation score from control group have lower variance and stronger normality than the methyltransferase-treated group(Supplementary Note, Supplementary Fig.3D-E).

External Modification Confoundings in Non-Open Chromatin Affect Detection: Except for discrete data-adaptive comparisons used by MAGNIFIER, we further designed an alternative data-adaptive model based directly on continuous methylation levels (Supplementary Method). However, this approach risked higher false positives (Supplementary Fig.3A) which mainly due to reads with non-open chromatin specific exogenous methylation (Supplemnetary Fig.3B-C). To assess the impact of outlier data on our model, we conducted a data simulation as detailed in the Supplementary Method. When the case data contained 10% outlier data points (3 out of 30), the continuous model's estimates for open chromatin accessibility scores at the locus increased progressively with the rising mean of the outlier data distribution(Supplementary Fig.3F). This pattern suggests that methods based on likelihood ratio statistics are particularly susceptible to the influence of a small number of outliers. With advancements in basecalling models leading to a larger differentiation between scores obtained for methylated versus unmethylated modifications, the continuous data-adaptive model is expected to face an increased risk of false positives due to non-open-chromatin-specific exogenous modification confoundings. Conversely, the discrete data-adaptive method, relying on Fisher's exact test, adapts a dynamic threshold from the control data distribution to discretize control and case data into binary variables. The incorporation of outliers in the case data, reflecting a $Z^1_{i,j,k} = 1$ state (Supplementary Fig.3E), does not affect MAGNIFIER's ability to accurately identify the open or non-open state ($B_{i,k}$) of target sites when the proportion of such mixing is low.

Data-Adaptive Methods Mitigate the Impact of Endogenous Methylation Modifications: To evaluate the data-adaptive method's effectiveness in removing the influence of endogenous methylation confoundings, we

further simulated scenarios of low-level endogenous methylation by fixing the methylation levels in the case data at 80% and 90%. We observed that when the level of endogenous methylation was 15% or lower, the data-adaptive method could successfully detect methylation differences between case and control data,(Supplementary Fig.4H).
The incorporation of multiple base markers significantly boosts MAGNIFIER's capacity to discern open chromatin regions. Taking the genomic segment chr1:40,970,000-40,990,000 in the K562 cell line as an example, chromatin regions undetectable with solely 6mA information become identifiable through the combined application of 6mA, GpC, and CpG markers, (Supplementary Fig.5A). A critical reason behind this improvement is the under-representation of certain base markers; specifically, regions of open chromatin with a lower proportion of "A" bases are overlooked in 6mA modification data, (Supplementary Fig.5C).

Supplementary Figures

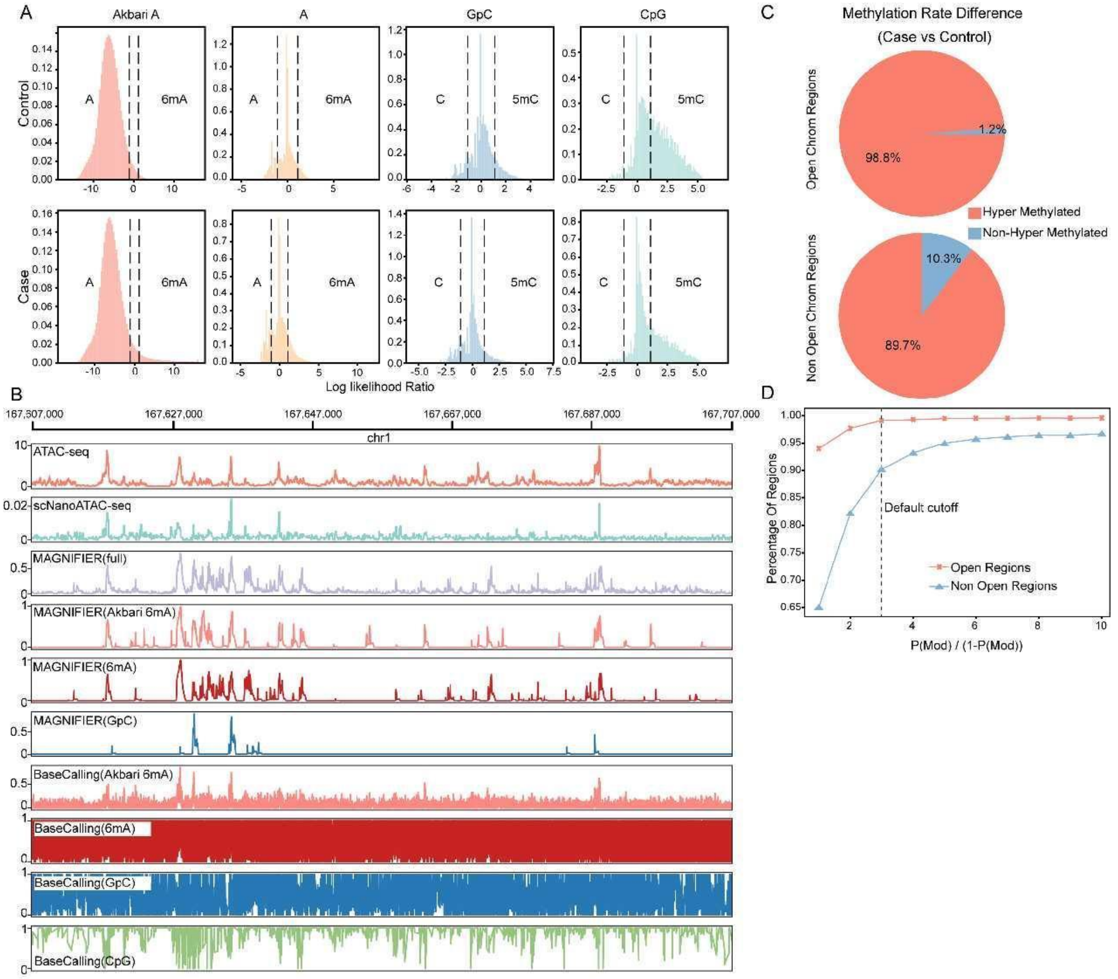

Supplementary Fig.1. The influence of base calling errors in open accessible chromatin finding. A. Histogram shows the distribution of Methylation score of 6mA called by Akbari's basecalling model (light red), 6mA calculated by default model (light yellow), GpC methylation (light blue), and CpG methylation (light green). B. Snapshot of region chr1:167,607,000-167,707,000. Result of different methods were labeled at each data track. C. Pie charts show the difference of modification level between methyltransferase-treated and methyltransferase-untreated K562 sample. D. Line plot show the percentage of regions with higher mean methylation level in methyltransferase treated sample than untreated sample under different modification cutoffs.

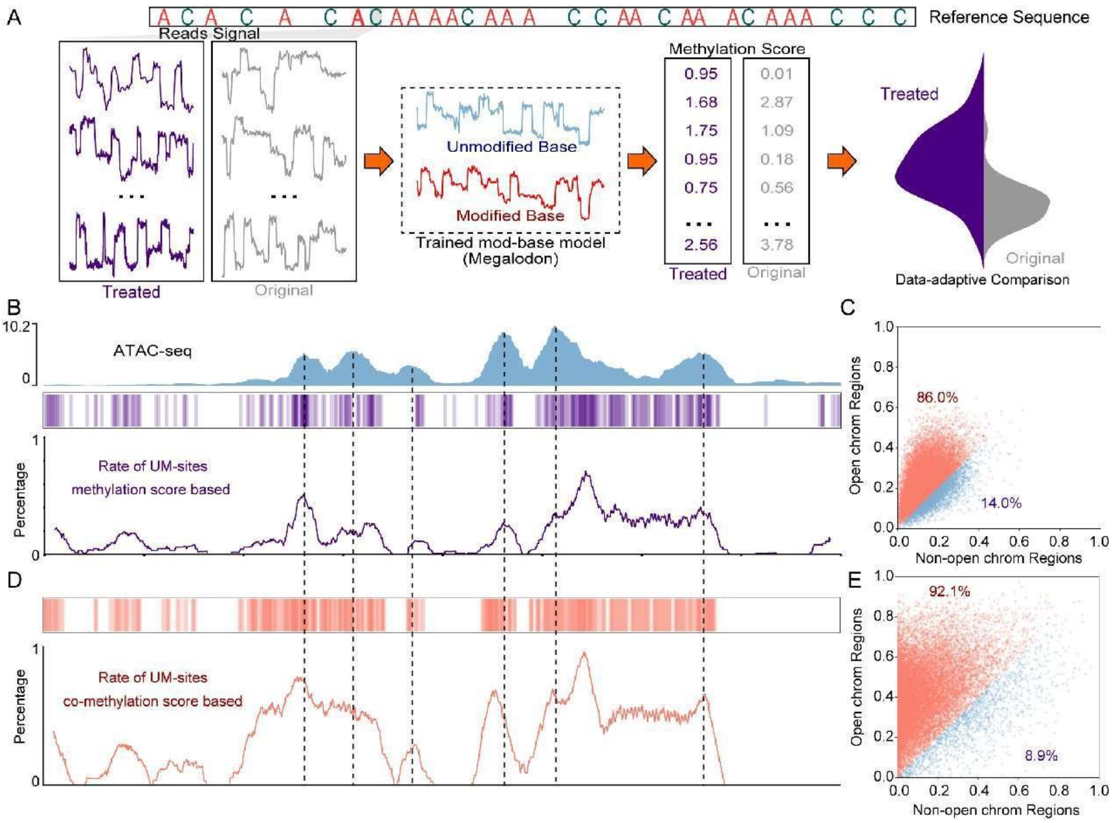

Supplementary Fig.2. Data-adaptive comparison and local co-methylation score help recognizing open accessible chromatin regions.

A. Overview of the data-adaptive comparison idea.

B. A snapshot shows accessible regions and inaccessible regions defined by ATAC-seq. Significantly exogenous modified bases (UM-sites) based on methylation score comparison are labeled in purple bars at the middle of plot ($p<0.01$, fisher-exactly test). The local UM-site rate distribution plot is placed at the bottom

C. Scatter plot shows rate of UM-site, based on methylation score comparison, in inaccessible regions (Non Open Region) and matched accessible regions defined by ATAC-seq (Open Region). Peak-Flank pairs with hyper UM-rate in peak were marked in red; Peak-Flank pairs with hypo UM-rate in peak were marked in blue;

D. A snapshot shows accessible regions and inaccessible regions defined by ATAC-seq. Significantly exogenous modified bases (UM-sites) based on co-methylation score comparison are labeled in red bars at the middle of plot ($p<0.01$, fisher-exactly test). The local UM-site rate distribution plot is placed at the bottom.

E. Scatter plot shows rate of UM-site, based on co-methylation score comparison, in inaccessible regions (Non Open Region) and matched accessible regions defined by ATAC-seq (Open Region). Peak-Flank pairs with hyper UM-rate in peak were marked in red; Peak-Flank pairs with hypo UM-rate in peak were marked in blue;

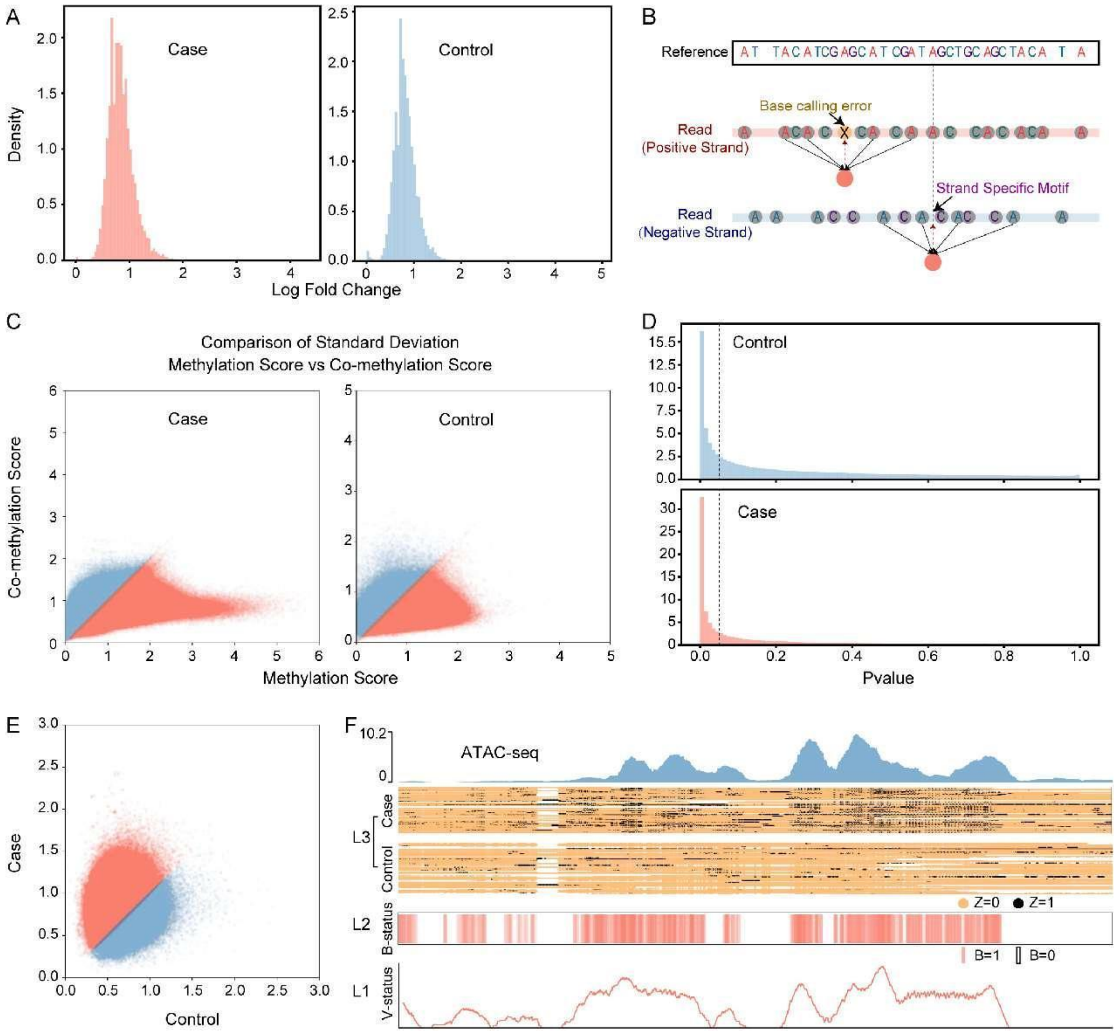

Supplementary Fig.3. Data properties and calculation detail of latent layers.

A. Histogram shows the fold change of included sample size between strategy leveraging local co-methylation score and point-by-point methylation score. The data of methyltransferase-treated are colored in red, and methyltransferase-untreated in blue.

B. Schematic shows the detail of co-methylation score calculation for bases with base calling error and strand specific condition.

C. Scatter plots show the distribution of the standardard deviation value of each candidate base between strategy leveaging local co-methylation score and point-by-point methylation score from methyltransferase-treated and methyltransferase-untreated samples. Bases with lower standard deviation values under co-methylation strategy are colored in red, the others in blue.

D. Histograms show the distribution of pvalue based on shapiro-test for data points from methyltransferase-untreated (blue) and methyltransferase-treated sample (red). Doted line represents p=0.05.

E. Scatter plot shows the standard deviation values of bases located within accessible regions defined by ATAC-seq from methyltransferase-untreated samples (x-axis) and methyltransferase-treated (y-axis). Data points with higher standard deviation in methyltransferase-treated group are colored in red, the others are colored in blue.

F. A snapshot shows the variable distribution of model layer 1-3 in the example region same as Fig.2B. Color labels of Layer 3 and Layer 2 are as shown on the legends.

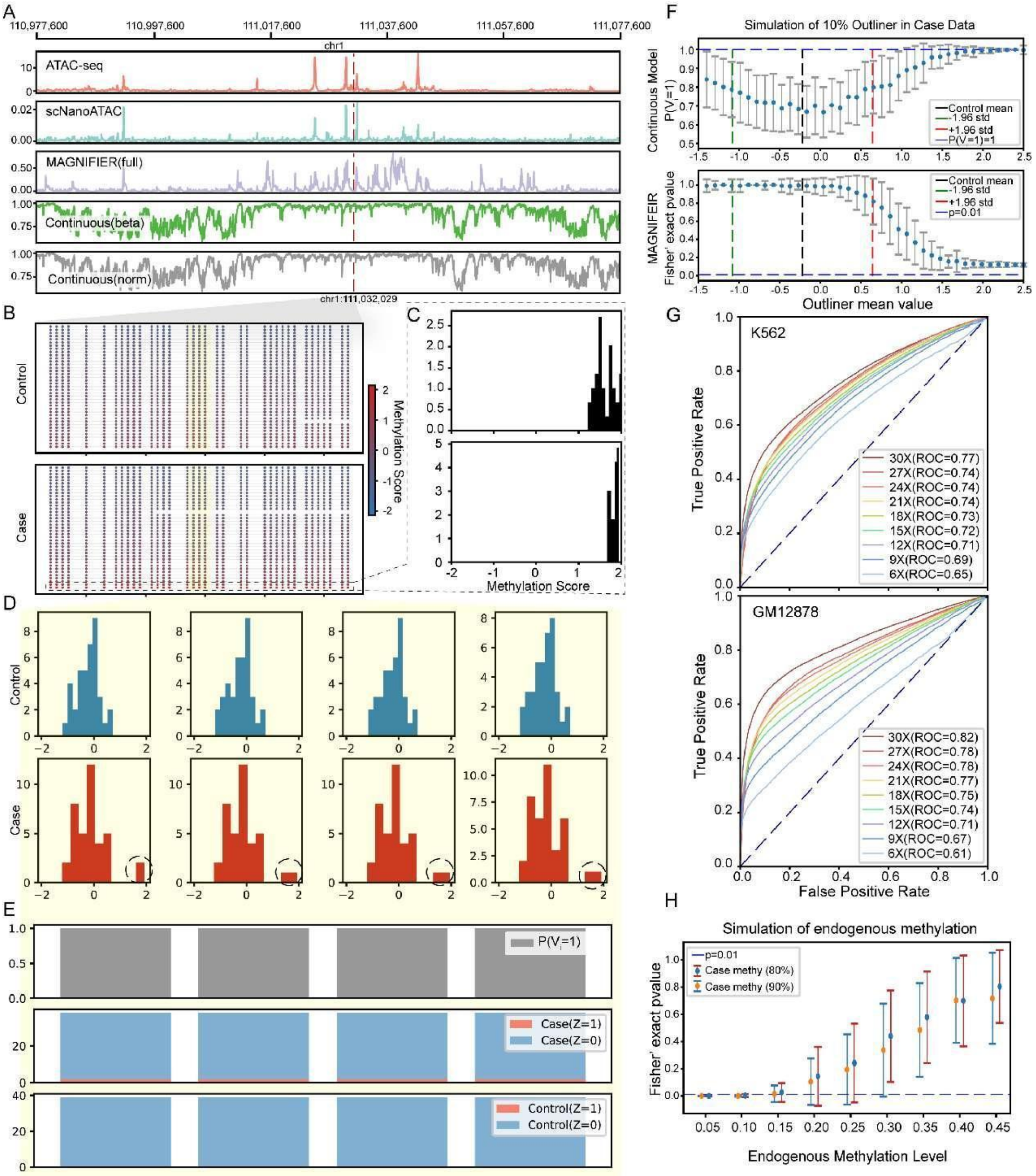

Supplementary Fig.4. The influence of non-open-chromatin-specific exogenous methylation and endogenous methylation. A. Snapshot shows ATAC,scNanoATAC, MAGNIFEIR and two continuous data-adaptive model output of region chr1:110,976,000-111,077,600. B. Dot plot shows the detailed methylation score of covered reads on position chr1:111,032,029. C. Histogram show the distribution of methylation score of A position from two outliner reads. D. Histograms show the distribution of methylation score for position nearby chr1:111,032,029. Samples from Control are colored in blue, and Case in red. E. Bar plot shows the out put of Continuous data-adaptive method, MAGNIFEIR Z status of Case data, and Control data. F. Error bar plot show the performance of Continuous data-adaptive method and MAGNIFIER's data-adaptive method in simulation data. G. ROC plot shows the performance of MAGNIFIER in detecting chrom1 open chromatin regions of K562 and GM12878 under different sequencing depth. H. Error bar plot shows the performance of MANIFIER's data-adaptive method in simulation data under different endogenous methylation level.

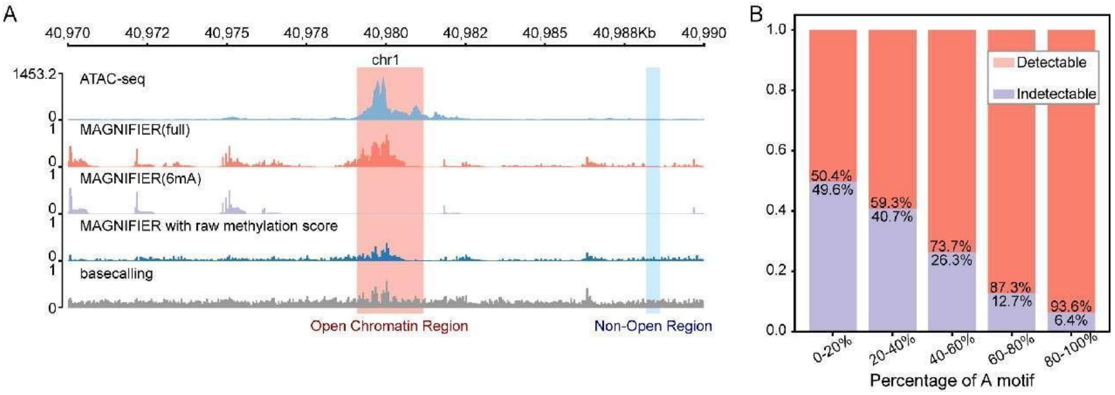

Supplementary Fig.5. Advantage of detecting open chromatin regions with multiple base markers. A. Snapshot of genome browser shows the overview chromatin accessible signal of different methods. The major open accessible region and example inaccessible region are high lighted with red and blue shadows repectively. B. Barplot shows the proportions of 6mA signal detectable accessible regions under different content of A bases.

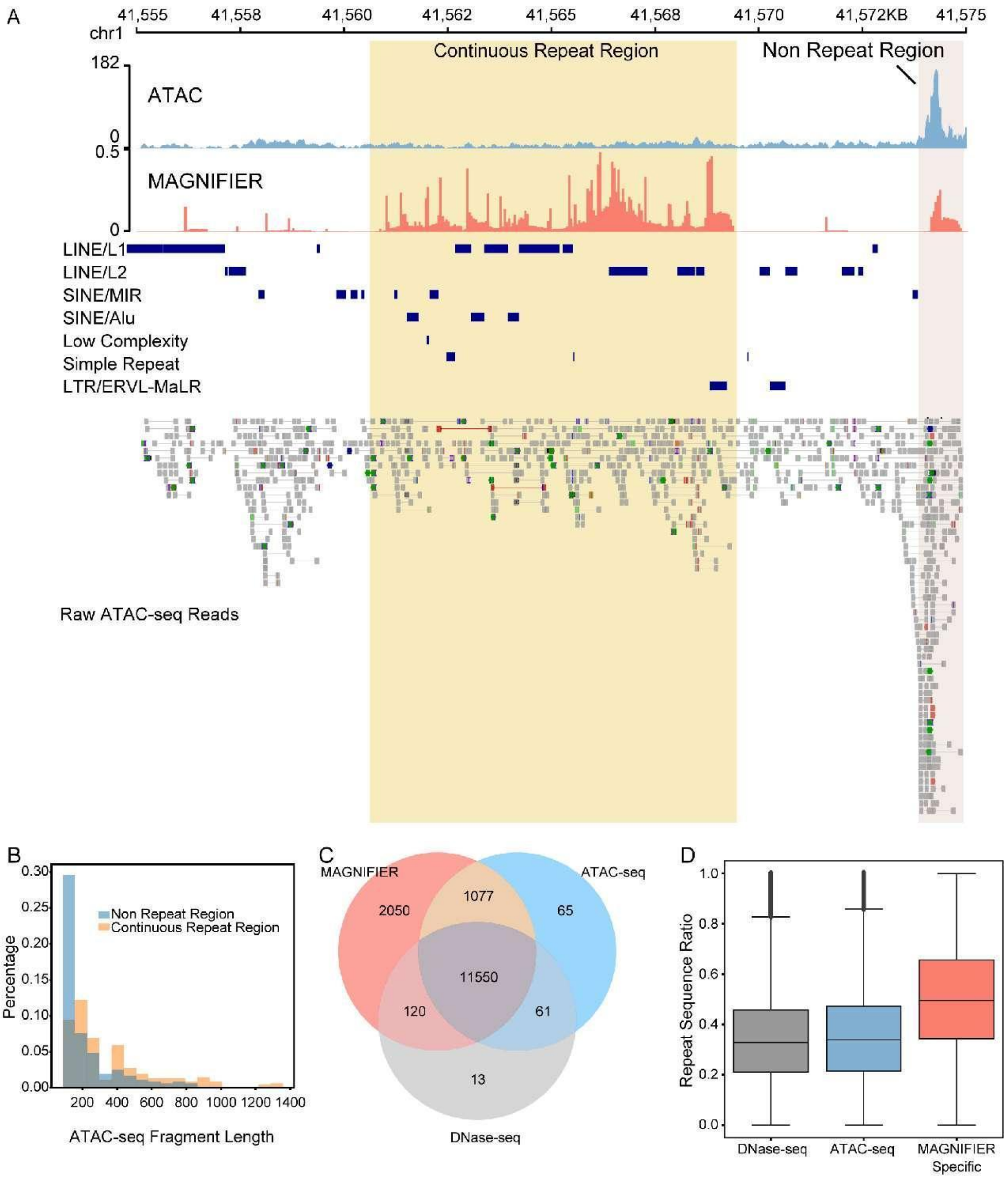

Supplementary Fig.6. Features of newly discovered open chromatin regions with MAGNIFIER.

A. snapshot shows the signal of ATAC-seq and MAGNIFIER in genome region with (yellow highlighted) and without (grey highlighted) repeat element. B. Histogram shows the length distribution of ATAC-seq fragment aligned within repeat genome region (Yellow) and non-repeat genome region (blue). C. Venn plot shows the number of transcript initial inaccessible regions with accessible chromatin defined by MAGNIFIER (red), ATAC-seq (blue) and DNase-seq (grey).

D. Boxplot shows the percentage distribution of repeat content for accessible transcript initial flank detected by DNase-seq (grey), ATAC-seq (blue) and specifically by MAGNIFIER (red).

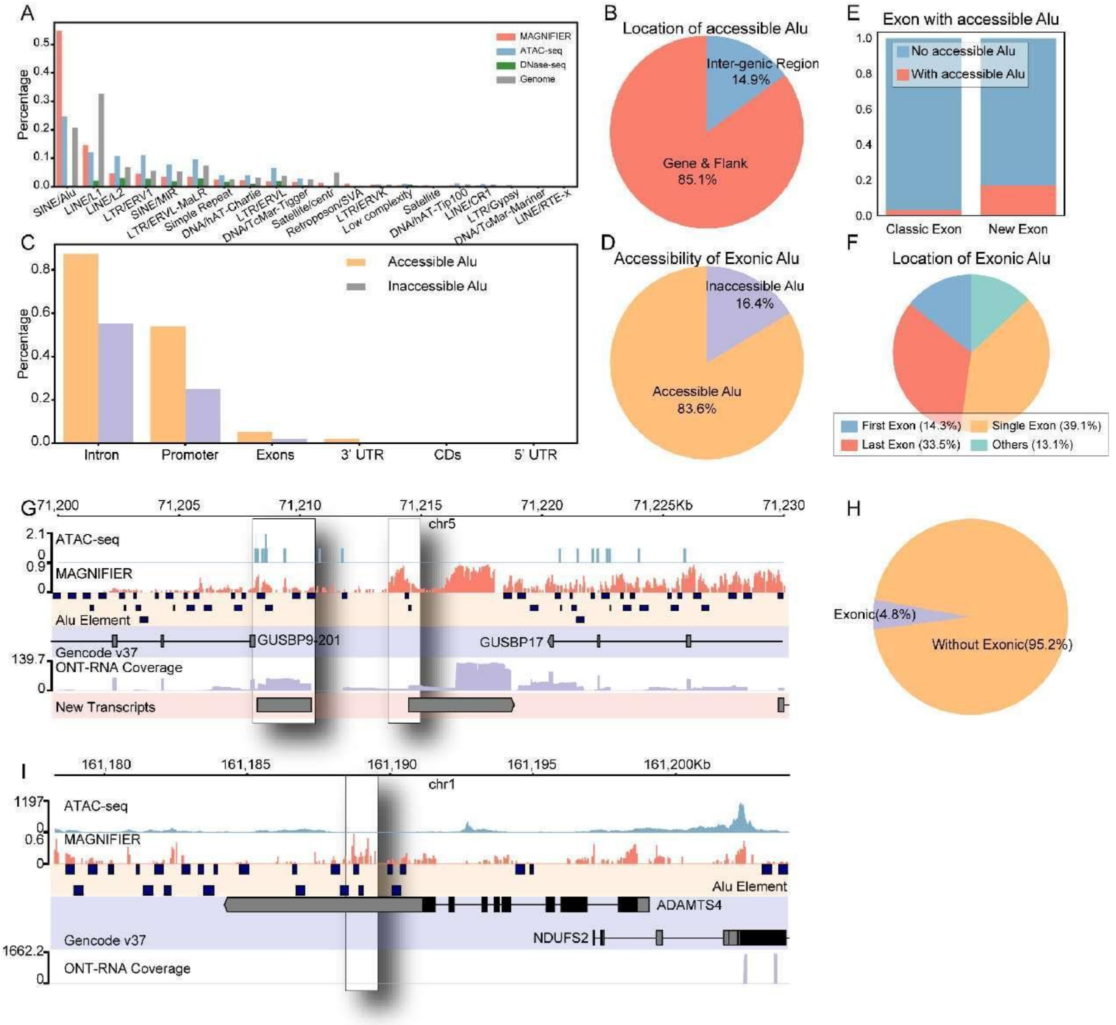

Supplementary Fig.7. The location of open accessible Alu element.

A. Barplot shows the percentage of MAGNIFIER(red), ATAC-seq (blue), and DNase-seq (green) defined accessible region with specific repeat elements. Genome background are colored in grey.

B. Barplot shows the detailed distribution of accessible Alu element relative to different gene element.

C. Piechart shows the percentage of exonic Alu element with or without accessible chromatin.

D. Piechart shows the location of exonic Alu element.

E. A snapshot shows the example of exonic Alu element with accessible chromatin that is undetectable by ATAC-seq.

F. Piechart shows the percentage of exonic and no exonic open accessible Alu elements.

G. A snapshot shows the example of non-exonic Alu element with accessible chromatin that is undetectable by ATAC-seq.
